# Learning hand hygiene from the champions: Investigating key compliance facilitators among healthcare workers through interviews

**DOI:** 10.1371/journal.pone.0315456

**Published:** 2024-12-19

**Authors:** Charis von Auer, Magdalena Probst, Wulf Schneider-Bachart, Susanne Gaube

**Affiliations:** 1 Department of Infection Prevention and Infectious Diseases, University Hospital Regensburg, Regensburg, Germany; 2 UCL Global Business School for Health, University College London, London, United Kingdom; Universiti Sains Malaysia - Kampus Kesihatan, MALAYSIA

## Abstract

**Introduction:**

Good hand hygiene compliance among healthcare workers is crucial for preventing healthcare-associated infections. While an extensive amount of research has focused on barriers to compliance with hand hygiene guidelines, there remains a critical gap in understanding the factors contributing to consistently excellent compliance among some individuals. Thus, the main aim of this study is to learn from these "champions" of hand hygiene and identify facilitating factors that enable and sustain high compliance using the Theoretical Domains Framework (TDF).

**Methods:**

In this qualitative study, we conducted problem-oriented semi-structured interviews with questions based on the 14 domains of the revised TDF. The *N = 25* participants included physicians and nurses from three German hospitals. They were selected based on a reported history of excellent hand hygiene compliance.

**Results:**

All topics discussed by the interviewees could be categorised into the 14 TDF domains. Five TDF domains were particularly prominent: *environmental context and resources*, *behavioural regulation*, *knowledge*, *social influences*, and *skills*. The single most important facilitator for good hand hygiene compliance among both physicians and nurses was the construct/code *goals* (i.e., patient protection and self-protection). Additionally, for physicians, developing hand hygiene as a *habit* was considered particularly advantageous. Conversely, nurses emphasised that learning correct hand hygiene during their vocational training was especially beneficial for good compliance.

**Conclusions:**

The results highlight the importance of clear goals, habit development, comprehensive training, adequate resources, and a positive culture of communication in promoting good hand hygiene practices. The TDF has been proven to be a suitable model for identifying facilitating factors for hand hygiene compliance among healthcare workers.

## Introduction

Worldwide healthcare-associated infections (HAIs) lead to increased patient morbidity and mortality and pose a substantial financial burden to health systems [1]. Adequate hand hygiene behaviour has shown to be a crucial factor in preventing HAIs, with sanitising using an alcohol-based hand rub being particularly effective [2, 3]. Above all, hand hygiene among healthcare workers (HCWs) plays a decisive role in preventing the transmission of pathogens [4]. For this reason, the World Health Organization (WHO) developed a universally applicable approach to establishing good hand hygiene among HCWs [5]. One aspect involves adhering to proper hand sanitation techniques to thoroughly clean the entire hands, which significantly reduces harmful pathogens [3, 5–7]. The other focuses on correctly identifying when hand hygiene is required. The WHO’s "5 Moments of Hand Hygiene" are used for training HCWs, assessing compliance, and developing interventions to improve adherence to guidelines [5]. Many interventions based on the WHO model have significantly improved compliance [8–10], but success is often short-lived, and behaviour change among HCWs is hard to sustain [11, 12]. To overcome these issues, many researchers have explored factors that hinder or promote hand hygiene, often using theories developed to understand behaviour change [13–17]. Theoretical models can help to understand facilitators and barriers to behaviour and to design and implement effective interventions [18, 19]. The Theoretical Domains Framework (TDF) is one of the most widely used and well-validated models for studying health behaviour in general and hand hygiene behaviour among HCWs in particular [16, 17, 20]. It was developed by behavioural scientists and implementation researchers with the goal of identifying the most relevant theory-based determinants of behaviour [20, 21]. These determinants were initially grouped into 12 domains [21] and regrouped into 14 domains in a revised version of the TDF [22]. The TDF domains with definitions are shown in Table 1.

**Table 1:** The 14 TDF domains with definitions as published by Cane et al. (2012).

Domains	Definition
1. Knowledge	An awareness of the existence of something.
2. Skills	An ability or proficiency acquired through practice.
3. Social/professional role and identity	A coherent set of behaviours and displayed personal qualities of an individual in a social or work setting.
4. Beliefs about capabilities	Acceptance of the truth, reality, or validity about an ability, talent, or facility that a person can put to constructive use.
5. Optimism	The confidence that things will happen for the best or that desired goals will be attained.
6. Beliefs about consequences	Acceptance of the truth, reality, or validity about outcomes of a behaviour in a given situation.
7. Reinforcement	Increasing the probability of a response by arranging a dependent relationship, or contingency, between the response and a given stimulus.
8. Intentions	A conscious decision to perform a behaviour or a resolve to act in a certain way.
9. Goals	Mental representations of outcomes or end states that an individual wants to achieve.
10. Memory, attention and decision processes	The ability to retain information, focus selectively on aspects of the environment and choose between two or more alternatives.
11. Environmental context and resources	Any circumstance of a person’s situation or environment that discourages or encourages the development of skills and abilities, independence, social competence, and adaptive behaviour.
12. Social influences	Those interpersonal processes that can cause individuals to change their thoughts, feelings, or behaviours.
13. Emotion	A complex reaction pattern, involving experiential, behavioural, and physiological elements, by which the individual attempts to deal with a personally significant matter or event.
14. Behavioural regulation	Anything aimed at managing or changing objectively observed or measured actions.

According to the TDF, any health behaviour, including hand hygiene, will be largely determined by these domains. However, depending on the specific behaviour, some domains may be more relevant than others [22]. Research suggests that interventions developed according to behaviour change theories, including the TDF, result in a more substantial improvement in hand hygiene than non-theory-based interventions [17, 23, 24]. However, even theory-based hand hygiene interventions have limitations, and there is still no clear solution for maintaining good compliance in the long term [12, 19, 25].

To date, research has mainly focused on identifying reasons for and overcoming barriers to inadequate compliance with hand hygiene guidelines among HCWs, with many systematic reviews assessing why failures occur and the effectiveness of interventions to improve adherence [8, 9, 16, 19, 26, 27]. Although studies on effective compliance and best practices exist [28–30], the focus remains on addressing shortcomings rather than understanding factors leading to good adherence. Some HCWs continuously exhibit excellent compliance, yet very little attention has been paid to understanding the conditions that enable and sustain their actions. Research has shown that learning from people who follow best practice principles can be helpful as these individuals have often developed effective methods and strategies to achieve their behavioural goals, offering valuable insights for others to benefit from [31, 32]. At present, it is unclear how and why HCWs who continuously show excellent hand hygiene compliance have reached this point. Thus, the main aim of the present research is to identify facilitating factors leading to and sustaining high compliance based on the TDF model. Furthermore, nurses generally demonstrate better hand hygiene compliance than physicians [11, 25, 33]. Consequently, the second objective was to determine whether different factors are relevant to promoting good hand hygiene compliance among these two professional groups. The information gathered should help optimise current and future behaviour change interventions to improve hand hygiene behaviour among HCWs who still struggle to meet standards and ultimately prevent further infections.

## Methods

### Research design

With this qualitative study, we aimed to investigate factors that positively influence the hand hygiene behaviour of HCWs and to examine whether different aspects are relevant to physicians and nurses. Hence, HCWs from three German hospitals were invited to participate in problem-oriented semi-structured interviews. For this purpose, we developed a structured interview guide with questions based on the 14 TDF domains [22]. The interview guide was pretested with two nurses to ensure comprehensibility. The study design was approved by the Research Ethics Committee at the University Hospital Regensburg (#22-2953-101).

### Recruitment and participants

Participants were selected based on a history of excellent hand hygiene compliance assessed by the WHO’s "Five Moments for Hand Hygiene" model [5]. Infection prevention and control (IPC) staff from the three hospitals facilitated contact with potential interview candidates, who were identified through direct process observation. The observations are conducted annually across various wards in each hospital. They are announced by the IPC teams and follow the guidelines established by the “Aktion Saubere Hände,” a nationwide campaign to improve hand hygiene compliance in German healthcare institutions [34]. It is based on the WHO’s campaign Clean Care is Safer Care. Compliance with the 5 WHO indications for hand hygiene is observed and documented according to the established process and protocols, which are harmonised across all participating healthcare facilities [34]. If observers noticed that a healthcare worker demonstrated excellent compliance, they requested permission from that individual to forward their name to the study investigators. The participant recruitment started on June 29th and ended on October 11th of 2022, with the last interview being conducted on October 26th. A total of 33 HCWs were invited to participate in this study, of which 27 HCWs agreed to be interviewed (participation rate = 0.82), including 10 physicians and 17 nurses from seven departments/specialities (see Table 2). To ensure data privacy, HCWs’ age and working experience were not assessed. Among the physicians, nine (90%) identified as male, and in the group of nurses, six (35%) identified as male. One physician and one nurse were excluded from the analysis since their interview responses indicated they might not consistently demonstrate excellent hand hygiene compliance. Consequently, we were left with a final sample of *N* = 25.

**Table 2:** Number of interviewed participants and their respective departments/ specialities.

Department	Physicians	Nurses
Intensive Care Unit	4^*^	11^*^
Urology	1	0
Obstetrics / Gynaecology	0	3
Transfusion-Medicine	1	0
Nuclear medicine	1	0
Radiotherapy	0	1
Internal Medicine	3	2

Note: *The table contains the 27 interviewed HCWs. One physician and one nurse were excluded from the analysis.

### Data collection and procedure

Data for the cross-sectional study were collected during the problem-oriented semi-structured interviews. This type of interview is well-suited for theory-driven research [35]. All interviewees received the same questions from the TDF-based interview guide, along with optional follow-up questions or prompts if needed. Interviews were conducted in person or online via Zoom (Zoom Video Communications, Inc.) between June and October 2022. On average, the interviews lasted *M* = 20.9 minutes (*SD* = 6.7, *min* = 11.9, *max* = 35.5) and were conducted in German. At the beginning of the interviews, participants were informed about the purpose and procedure of the interview and that participation was voluntary and could be terminated at any time. All interviewees gave written consent to participate in the study. The interviews were recorded and transcribed using Trint (Trint Limited).

### Data analysis

The interview transcripts served as the basis for the data analysis with MAXQDA (VERBI Software). We conducted a content analysis, as this approach offers the possibility of qualitative and quantitative analysis of the transcribed interviews. A combination of inductive and deductive analysis, guided by the steps described by Fereday et al. [36], was used. First, we developed an initial codebook based on the TDF domains and constructs (S1 File). All constructs were converted into codes to be applied to the transcripts. The codebook contains the names of the codes with a definition and a description of how to apply them to the transcripts. Some constructs occur simultaneously in different TDF domains. To ensure better workability, each construct was exclusively assigned one domain (see codebook). One exception was made for "feedback," which was assigned to both *social influences* and *behavioural regulation*. The concept is integral for both domains. Moreover, there is a difference between feedback provided directly by a person and feedback provided indirectly through data such as compliance statistics. Additionally, some other constructs/codes were adjusted or merged to improve workability and simplify coding. All adjustments are recorded in the codebook. Before coding, two authors read and summarised all transcripts to extract the key points. New themes that emerged during this step were added as codes to the codebook and assigned to the appropriate TDF domain and construct. If necessary, a new construct was also added. Next, the transcripts were analysed by applying the codes from the codebook to identify meaningful units of text. Full sentences were chosen as the smallest unit of coding. Different codes could be applied to the same sentence or section. Each transcript was coded independently by two coders (CvA and MP), who then compared their results. If the coders disagreed on a code after a discussion, a third author (SG) facilitated a consensus. During the coding process, additional inductive codes were defined and assigned to segments that described a new topic. If the new code could not be assigned to an existing construct, a new construct was also added. Notably, some of the new constructs had already been part of the original version of the TDF [21]. Also, some new codes simply expanded on codes from the initial codebook, in which case new subcodes were added. As a result, constructs and codes overlap strongly but do not always have to match. In addition, not all TDF constructs were relevant for coding and, therefore, did not become a code. For further analysis, a table was created to provide an overview of the codes and the number of times they were mentioned in total and split by physicians and nurses (S1 Table). Further supplements, including the preregistration and interview guide can be found in the project’s online repository (https://osf.io/39ybf/)

## Results

The codebook contains 193 codes (99 principal codes and 96 subcodes), which were applied to the transcripts. Surprisingly, we did not identify codes outside of the original 14 TDF domains. Coding the 25 transcripts yielded 2521 applied codes, averaging 100 codes per transcript. In total, 1011 and 1510 codes were assigned to the physicians’ and nurses’ interviews, respectively. For further analysis, every code was categorised as either a positive influencing factor (facilitator), a negative influencing factor (barrier), or a neutral factor/not clearly attributable. These assessments were made by looking at the context in which the codes were mentioned, either as answers to specific questions or when interviewees directly labelled something as having a positive or negative impact. While 1921 codes were classified as facilitators, only 337 were considered barriers and 263 codes as neutral/not clearly attributable. This extreme distribution indicates that the interviewees primarily emphasised factors that positively impact hand hygiene compliance.

Subsequently, a more detailed discussion will address the TDF domains, constructs, and their corresponding codes. Given the extensive amount of data, the focus will be on factors frequently mentioned, limited to those with a cumulative occurrence of 25 or more codes across nurses and physicians combined. These codes are summarised in Table 3. Since more nurses than physicians were interviewed, the table lists not only the absolute numbers but also the relative proportion of mentions of a code per person to enable a better comparison between the professional groups (for nurses, the numbers were divided by 16, and for physicians, they were divided by 9).

**Table 3:** Most frequent codes (≥25) categorised as facilitators / barriers / neutral to hand hygiene behaviour within the TDF.

				Quantity of codes (≥25)				
Domains, constructs, and codes	Facilitator	Barrier	Neutral	Total	Physicians	Nurses	Relative* Physicians	Relative* Nurses
**Knowledge**				**242**				
• Knowledge	X			107	41	66	4.56	4.13
• Procedural knowledge	X			89	38	51	4.22	3.19
• Knowledge of the task environment	X			46	26	20	2.89	1.25
**Skills**				**203**				
• Skills development	X			131	38	93	4.22	5.81
▪ Training courses	X			28	11	17	1.22	1.06
▪ Vocational training (nurses)	X			30	0	30	0.00	1.88
**Social/professional role and identity**				**135**				
• Leadership	X			45	16	29	1.78	1.81
• Professional role	X			62	24	38	2.67	2.38
• Professional identity	X			28	10	18	1.11	1.13
**Beliefs about capabilities**				**50**				
• Execution is easy (Perceived behavioural control)	X			50	22	28	2.44	1.75
**Optimism**				**40**				
• Optimism	X			40	20	20	2.22	1.25
**Beliefs about consequences**				**121**				
• Consequences	X			121	52	69	5.78	4.31
▪ Negative consequences	X			60	31	29	3.44	1.81
▪ Positive consequences	X			61	21	40	2.33	2.50
**Reinforcement**				no code in this domain was coded over 25 times				
**Intentions**				no code in this domain was coded over 25 times				
**Goals**				**164**				
• Goals	X			164	68	96	7.56	6.00
▪ Protection of others	X			55	22	33	2.44	2.06
• Protection / Well-being of patients	X			41	17	24	1.89	1.50
▪ Self-protection	X			40	16	24	1.78	1.50
▪ Avoid transmission of germs	X			33	14	19	1.56	1.19
**Memory, attention and decision processes**				**152**				
• Attention	X			83	37	46	4.11	2.88
▪ Be attentive to it yourself	X			47	21	26	2.33	1.63
▪ Draw attention to it	X			36	16	20	1.78	1.25
• Memory	X	X		69	36	33	4.00	2.06
▪ Execution forgotten		X		32	20	12	2.22	0.75
▪ Remember the execution	X			27	13	14	1.44	0.88
**Environmental context and resources**				**355**				
• Facilitators	X			117	43	74	4.78	4.63
▪ Education, continuing education, training	X			39	13	26	1.44	1.63
▪ Resources / material resources	X			41	17	24	1.89	1.50
• Availability of disinfectant	X			27	14	13	1.56	0.81
• No barrier			X	51	17	34	1.89	2.13
• Barriers		X		162	64	98	7.11	6.13
▪ Resources / material resources		X		99	33	66	3.67	4.13
• Lack of time		X		51	20	31	2.22	1.94
• Person x environment interaction	X			25	14	11	1.56	0.69
**Social influences**				**208**				
• Supervision	X			37	15	22	1.67	1.38
• (Hierarchy / different profession) not relevant to feedback	X			26	10	16	1.11	1.00
• Feedback**	X			86	35	51	3.89	3.19
▪ Getting feedback	X			30	12	18	1.33	1.13
▪ Giving feedback	X			47	19	28	2.11	1.75
• Modelling	X			34	14	20	1.56	1.25
• Social pressure	X			25	9	16	1.00	1.00
**Emotion**				**69**				
• Disgust relevant	X			37	13	24	1.44	1.19
• Fear, anxiety relevant	X			32	13	19	1.44	1.19
**Behavioural regulation**				**276**				
• Action planning, implementation intention	X			26	8	18	0.98	1.13
• Workflow	X			46	15	31	1.67	1.94
• Feedback**	X			92	40	52	4.44	3.25
▪ Getting feedback	X			38	17	21	1.89	1.31
▪ Giving feedback	X			46	19	27	2.11	1.69
• Habit:	X			112	54	58	6.00	3.63
▪ Positive influence	X			49	23	26	2.56	1.63

Note. *Since more nurses than physicians were interviewed, the table lists not only the absolute numbers but also the relative proportion of mentions of a code per person to enable a better comparison between the professional groups (for nurses, the numbers were divided by 16, and for physicians, they were divided by 9). **While it has been a standard practice to code all double-appearing constructs in a single domain, an exception has been made for the construct/code "feedback".

### Most important themes/concepts within the most relevant domains

**Fig 1:**
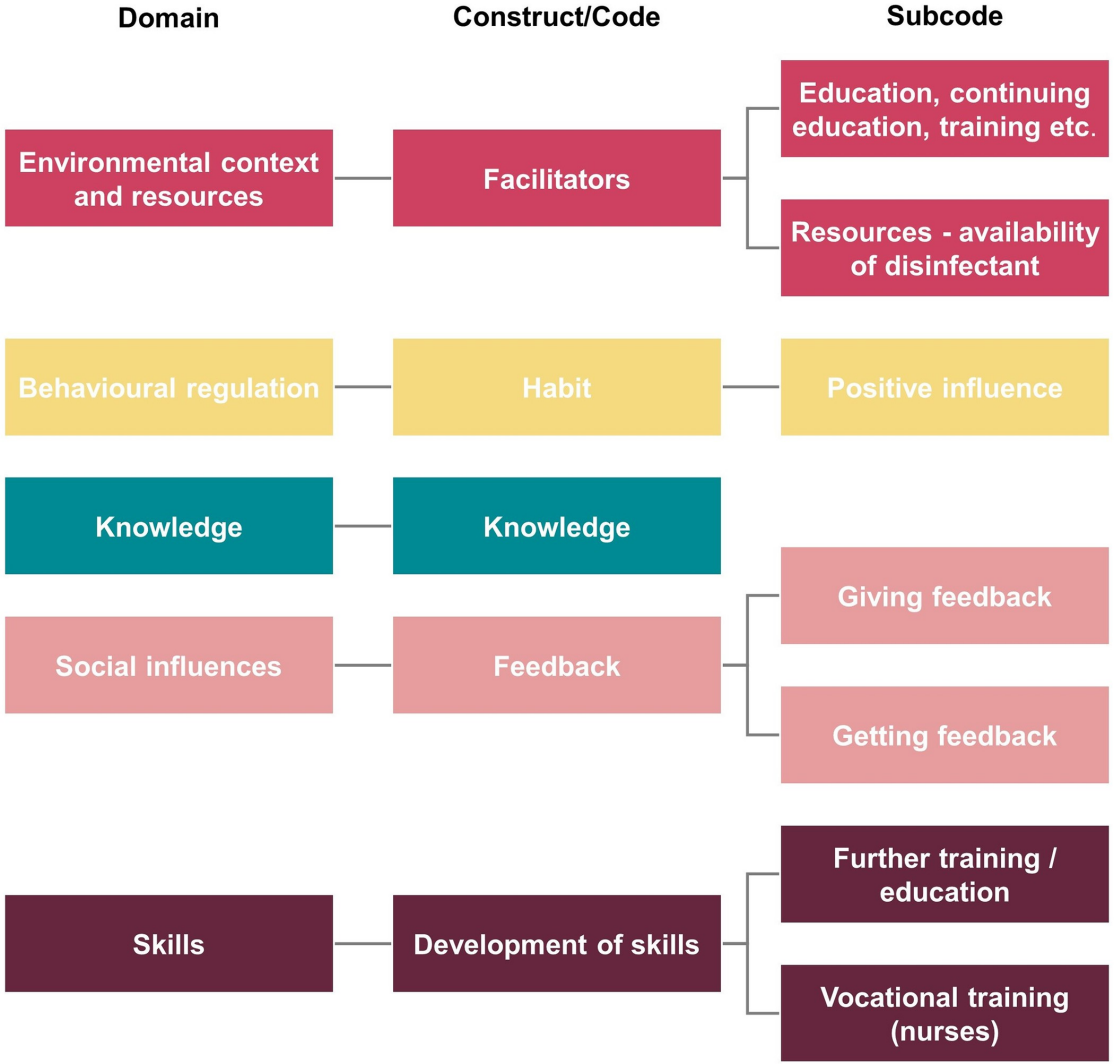
The top five most frequently coded domains with the most relevant construct/code.

As the main aim of the present research is to identify facilitating factors leading to and sustaining high compliance, we are focusing more on facilitators than barriers. The result order is based on the total number of mentions per domain in descending order. The top five domains with their most relevant associated construct/code are summarised in Fig 1.

The second objective is to determine whether different factors are relevant to promoting hand hygiene compliance among the different professional groups. For this purpose, the percentage difference between the average relative numbers of doctors and nurses was calculated ((P-N)/[(P+N)/2)]x100) and if we found a strong difference (≥50%), it will be highlighted which professional group has predominantly addressed the topic.

#### Environmental context and resources

In this domain, 193 facilitating factors but also 162 barriers were mentioned by the interviewees. The most frequently mentioned facilitators by both professional groups were good education/training and sufficient resources like enough time and personnel. Moreover, physicians emphasised the importance of having disinfectants and dispensers widely available.

“*So certainly conducive is the good availability. As I said, we actually have the dispensers mounted everywhere*”. [P-H1-#17] (P = physician / N = nurse; H = Hospital 1,2 or 3; #X = individual number assigned to each participant)

Very important, especially for physicians, was the interaction with their environment and surroundings, such as seeing signs about proper hand disinfection at sinks or having dispensers placed along the routes they walk by.

“*These pictures from the Campaign “Aktion Saubere Hände", where you can also see these graphics […]".* [P-H1-#9]

One notable obstacle consistently mentioned by the participants was a lack of time to engage in adequate hand hygiene.

“*Well, of course, the time pressure is a hindrance, that’s for sure. So you say I’ve just forgotten. It rings in the back, rings in the front, I don’t know where my head is’”.* [N-H1-#15]

#### Behavioural regulation

In this domain, only facilitators were mentioned by the interviewees as relevant topics. Respondents, especially physicians, most frequently mentioned that establishing proper hand hygiene as a habit in their work process is beneficial for compliance. They stated that disinfecting hands is part of their work routine and an action they automatically perform without having to think about it explicitly.

“*Yes, as I said, this is crucial. Transitioning this into second nature is actually the essence in order to follow through with it in everyday life”*. [P-H1-#19]

Participants also mentioned that predefined workflows and process descriptions that include when hand disinfection is required are beneficial.

“*So I think it’s totally important to have set workflows and also have set ways of doing things”.* [N-H3-#23]

Related to this, planning when to perform hand hygiene during a task and good self-organisation were considered helpful for adhering to the guidelines. For instance, some interviewees stated that they actively use the time while disinfecting their hands to plan the next steps of the procedure. Another strategy mentioned is to rigorously postpone other tasks to ensure proper hand hygiene.

“*So concrete strategies help for sure. And that you simply make a resolution to disinfect your hands every time you go into the patient’s room, regardless of whether you are doing something or not. And I think something like that actually helps*”. [P-H1-#20]

Both physicians and nurses see feedback as an important and available tool to promote proper hand hygiene. Notably, they talked more about the benefit of giving feedback to colleagues than about receiving it themselves. In addition to personal feedback, receiving aggregated data in the form of unit- or hospital-wide compliance statistics and compliance certificates, such as the one issued by the campaign “Aktion Saubere Hände” (a nationwide campaign to improve hand hygiene compliance), can also be helpful for assessing the current state and improving hand hygiene behaviour. Moreover, some of the interviewees expressed their perception that positive feedback was seen as praise or even a form of reward.

*“Furthermore, in the hospital-wide infection prevention and control board meeting, you receive a nicely prepared statistical analysis of how the whole surveillance looked like”.* [P-H1-#9]

#### Knowledge

Overall, the interviewees demonstrated a profound understanding of pathogen transmission processes, HAIs, their consequences, and the critical role of hand hygiene in mitigating transmissions and infections.

“*With the background that nosocomial germs are, of course, a huge problem in hospitals and that the survival of patients is affected. In other words, I believe that a high standard of hygiene is an essential part of high-quality medicine*”. [P-H1-#18]

Individuals from both professions also showed robust procedural knowledge regarding the correct execution (how) and timing (when) of hand hygiene. For instance, one person listed all five moments of hand hygiene according to the WHO model. The interviewees consistently highlighted their awareness of the task environment, recognising the significance of thorough hand hygiene in the presence of vulnerable patients, within specific departments, and during certain tasks like dressing changes or central venous catheter handling.

“*What can also happen with our people is that if someone gets a multidrug-resistant germ and is severely immunosuppressed, then he already has a problem if he can no longer be treated, bone marrow transplanted or something like that*”. [P-H1-#18]

#### Social influences

The prevailing theme in this domain was direct feedback, primarily among colleagues. Participants predominantly focused on providing feedback when they witnessed incorrect hand hygiene behaviour among their peers and colleagues. Furthermore, they also expressed their openness and desire to receive feedback on both positive and negative performance from their colleagues or IPC staff during compliance observations.

“*Yeah, so I think it’s extremely important. […] I would call someone out on it if they don’t do it, but at the same time I expect them to call me out if I don’t do it*”. [P-H1-#14]

The interviewees predominantly stated that hierarchical disparities or belonging to a different professional group do not influence the likelihood and willingness to provide feedback regarding someone’s hand hygiene behaviour.

“*It doesn’t make any difference in the context of their profession because I would tell everyone, so from the head physician to the cleaning staff. If it’s just not correct or you can improve it, then I think it’s fine to address that*”. [N-H1-#7]

Another facilitator is being supervised or observed by others. The participants noted recurring compliance observations by IPC staff on their wards, which keeps IPC topics salient and, when accompanied with feedback, can aid in modifying incorrect behaviours. This is also true for being observed by one’s own colleagues, which can draw attention to improving hand hygiene.

“*And these observations are very important. When there are IPC experts on the ward, then this helps you*”. [P-H1-#14]

Interviewees mentioned that it can be beneficial to observe good hand hygiene behaviour from a role model and to follow their lead. Furthermore, they highlighted the importance of being a role model for others as a motivating factor for them to make a dedicated effort to perform adequate hand hygiene behaviour.

“*Role modelling is a key aspect that supports it. Just demonstrating a conscious ICP culture”.* [N-H1-#11]

Our participants stressed the importance of social pressure, in the form of expectations of patients, colleagues, and other hospital staff, in shaping hand hygiene behaviour. Additionally, they pointed out that when colleagues are observed practising hand hygiene, it can establish a social norm for others to emulate.

*“I think it’s important that patients have expectations. And it would be embarrassing for me personally if I were asked by patients that I should disinfect my hands. But that is good and that is certainly something that is also important for younger colleagues, an important incentive. The other way around? So if a colleague were to approach me now: ´Why didn’t you disinfect your hands?´ Of course, that would also be a point for me where I would say: ´I have to work on myself´”.* [P-H1-#17]

However, it is worth noting that some individuals expressed a contrary opinion, indicating that they do not experience social pressure regarding hand sanitisation or at least that it does not affect their behaviour.

*"So the expectation from the patients deliberately has no direct influence".* [N-H1-#11]

#### Skills

When asked about how and where interviewees developed their skills to perform correct hand hygiene behaviour, nurses frequently cited their vocational training as the primary learning opportunity.

“*Yes, classically in the 3-year training. That’s where you learned it in any case*”. [N-H1-#15]

Among physicians, regular on-the-job seminars were the most important source for skill development, rather than university courses. Nurses also frequently mentioned regular training in the workplace as important for developing competencies.

“*And then, of course, as a young resident, I participated in many advanced training courses in collaboration with the ICP. I learned a lot from that”.* [P-H1-#14]

#### Goals

Participants expressed a variety of goals associated with correct hand hygiene. The one most commonly mentioned by both professional groups was protecting others, especially their patients. Another significant goal was self-protection and the prevention of pathogen transmission in general.

*“Because, after all, I don’t want to contribute to anything being passed on in terms of infections or that I’m harming the patient”.* [N-H1-#7]

#### Memory, attention and decision processes

The interviewees affirmed their commitment to being mindful of their own hand hygiene behaviour and to actively practising hand disinfection.

“*Yes, for example you do it maybe more consciously before going on your break and you go to lunch. I do it even more consciously when I’m changing the dressing*”. [N-H3-#26]

However, they also find it beneficial when something draws attention to it. These cues include encountering signs regarding hand disinfection, being reminded by colleagues or undergoing compliance observations on the ward. The interviewees emphasised the importance of making hand hygiene a prevalent and salient topic in the hospital.

“*We did the compliance observations. We put up posters on how hand disinfection is done and why it is important. This is already a sustained topic and also very present in our facility. […]*”. [N-H2-#22]

Several individuals, especially physicians, mentioned that is generally easy for them to recall when they need to perform hand hygiene behaviour in compliance with the WHO model.

*"Also, it is important to remind yourself constantly. But in principle, I think it is easy”.* [P-H1-#14]

Nonetheless, they acknowledged occasional lapses in hand disinfection, particularly before patient contact, often attributed to high workload and stress.

*“That’s when it can sometimes go under, when you have already been called and you have to go to the operating room, but you are still in the last patient room“.* [P-H2-#25]

#### Social/professional role and identity

Many interviewees hold leadership positions such as attending physicians, head nurses, practical instructor, or link nurse. They emphasised that practising good hand hygiene is a part of their professional role as a leader.

“*Maybe it has to do with being a practical instructor, that you are responsible for the practical training of the students. You then have a role model function and it’s also more expected from you”*. [N-H3-#26]

Both physicians and nurses agree that adhering to the WHO model is an important responsibility and duty as HCWs and which is perceived by them as a matter of course.

“*We have a responsibility here […] that we don’t pass on pathogens”* [N-H1-#4] and *“But [good hand hygiene] is actually my own ambition when it comes to patient care”.* [P-H1-#1]

#### Beliefs about consequences

Understanding the consequences and repercussions of one’s actions play a decisive role in shaping hand hygiene behaviour. The interviewees expressed diverse beliefs regarding the outcomes of both good and bad hand hygiene practices. Notably, physicians appeared particularly aware of the adverse consequences of non-compliance.

“*If I don’t behave correctly in that regard, then yes, I may have done something great with a great surgery or a great procedure or aiding the patient, but I may have created a huge amount of damage if I didn’t follow hand hygiene".* [P-H1-#9]

In contrast, nurses placed greater emphasis on the positive outcomes associated with adhering to hand hygiene guidelines.

*“That’s why I believe that hand hygiene protects the patient, but also myself”.* [N-H3-#27]

#### Emotion

When discussing emotions that potentially could influence hand hygiene behaviour, participants noted that feelings of disgust towards bodily fluids and excretions serve as a motivating factor for them to clean their hands more frequently.

*"Yeah, I would put disgust at the top of the list. So that’s, I think, the biggest impetus for someone to immediately do massive cleaning and disinfection. […]".* [N-H1-#5]

Additionally, the fear of getting infected or infecting others appears to lead to better hand hygiene practices.

*"But that may be fear of contagion as a motivator".* [P-H1-#17]

#### Beliefs about capabilities

When asked if they believe they can consistently perform proper hand hygiene, the interviewees mostly expressed confidence, since they find it easy to execute the behaviour and know when to act.

*“When it comes to activities on the patient, that is, before and after. In the care itself, in the basic body care I am also very certain“.* [N-H3-#26]

#### Optimism

Notably more physicians than nurses expressed optimism about the potential for long-term improvement in hand hygiene behaviour among HCWs. Some respondents attributed this to the fact that they have observed significant improvements over the last few years.

*"Generally optimistic, in fact. I think this new generation, in particular, is paying more attention to this, especially in terms of the last Corona years. I think there’s been a bit of a change. Yeah, so I am optimistic".* [N-H1-#12]

### Summary of facilitators

**Fig 2:**
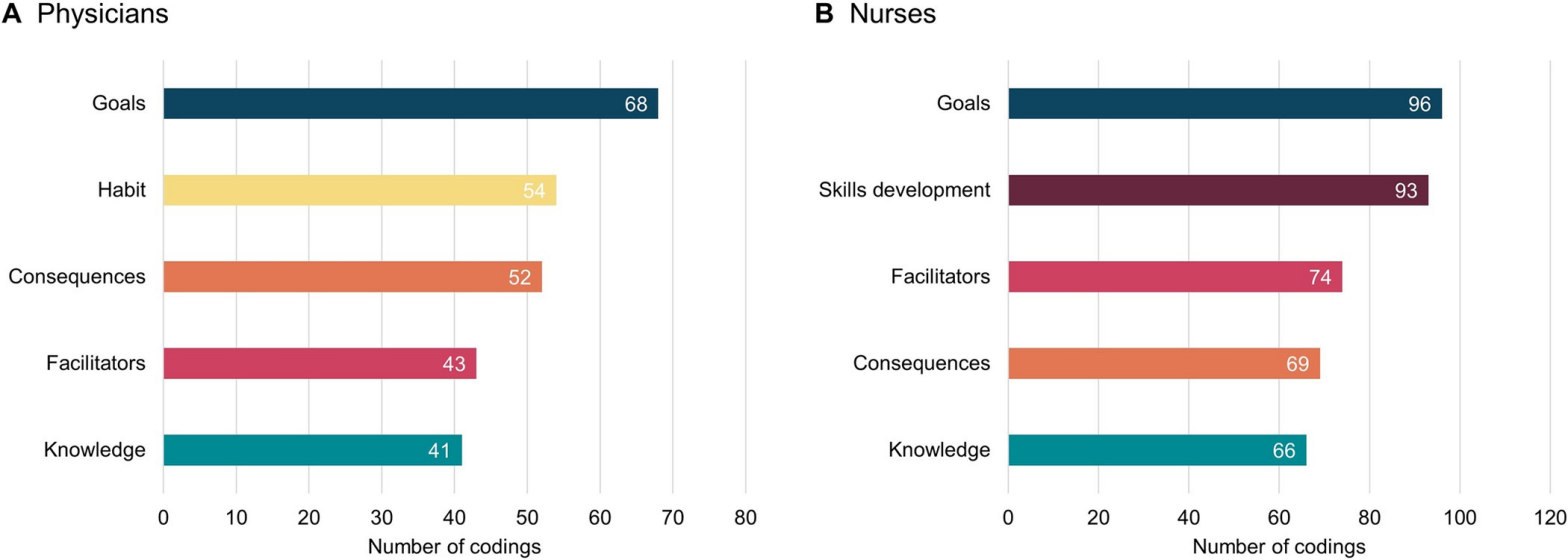
Top five most frequently coded facilitators for physicians and nurses.

Fig 2 summarises the top five most frequently coded facilitators that enable good hand hygiene compliance among physicians and nurses. Overall, the results for the two professional groups are very similar. The most frequently mentioned facilitator among both groups is the desire to achieve hand hygiene-related goals (i.e., protecting others and self). However, the second most important facilitator differs between the two occupations. Physicians attribute the success of maintaining high hand hygiene compliance to the fact that the behaviour has become a habit. Most nurses credited their high performance to skills acquired and developed during their professional training. The other important facilitators for both physicians and nurses are their beliefs about the consequences of good and bad hand hygiene, environmental facilitators such as sufficient availability of disinfectant, and their overall knowledge about pathogen transmission and HAIs and the importance of correct hand hygiene.

## Discussion

The findings of this study complement existing research on HCWs’ hand hygiene by taking a different approach and focusing on factors that promote good compliance rather than those associated with non-compliance. The interviewees discussed various topics assignable to the 14 TDF domains, indicating that the TDF is a suitable model for investigating determinants of hand hygiene behaviour among HCWs. A large proportion of the content fitted in the five domains: *environmental context and resources*, *behavioural regulation*, *knowledge*, *social influences*, and *skills*. Notably, the single most cited facilitator helping both physicians and nurses to maintain good hand hygiene was having clear goals, such as protecting patients and themselves. For physicians, establishing hand hygiene as a habit was also considered particularly advantageous, while for nurses, learning correct hand hygiene behaviour during vocational training was seen as helpful. This implies that establishing correct processes early in practical training programs and encouraging consistent practice and repetition might be key strategies for making hand hygiene an automatic behaviour.

Although the focus of this study was on the factors that facilitate good compliance, some barriers were mentioned even by our champions. Interestingly, some typical barriers that are often cited in the literature did not seem to be a problem for them, and even when confronted with those obstacles, they found ways around them. For example, previous research has established that key barriers to hand hygiene compliance among HCWs include environmental factors such as poor availability of disinfectant, skin irritations, wearing gloves, lack of time, and high workload [17, 27, 37]. While some of these issues, such as poor availability of products and aggressive products, did not appear to be a common problem in our interviewees’ workplaces, high workloads and time pressure were widespread. However, some of our champions found workarounds, such as actively using the rub-in time to plan their next steps.

In terms of facilitating conditions, our findings support the assumption that environmental factors, such as good availability of disinfectants and reminders in the workplace, encourage compliance [8, 15, 38]. The interviewees emphasised the importance of having signs about proper hand disinfection at strategic locations, such as sinks and dispensers being placed along their walking routes. Previous research has also shown that education and training are important facilitators for good compliance, which was supported by the statements of our participants [8, 15]. Nurses in particular emphasised the influence of their vocational training on their good hand hygiene behaviour. According to the HCWs, additional annual education and training programs also help to ensure that the five moments of hand hygiene are adhered to. Members of both professions demonstrated a comprehensive knowledge of HAIs and the importance of hand hygiene in preventing pathogen transmissions, which also has been previously shown to facilitate compliance [19]. In this context, it is beneficial for HCWs to understand the benefits of adequate hand hygiene, such as protecting both patients and themselves from infections [15, 17]. In addition, our findings show that physicians are especially aware of negative consequences associated with non-compliance, such as harming their patients. In line with previous research, we were able to show that having this knowledge, together with setting appropriate goals, promotes hand hygiene compliance [9]. Here, too, the protection of others (patients) and self-protection were the most important goals named by the interviewees. Notably, their commitment to protecting their patients was mentioned universally. Hand hygiene practices were also often linked to caring for vulnerable patients, such as immunosuppressed patients. This indicates that some participants view proper hand hygiene as an essential aspect of working in specialised areas. However, we did not recognise a notable difference in responses between HCWs from ICUs and other departments. Furthermore, social influences have been identified as important factors in improving hand hygiene compliance among HCWs, and our study underlines the importance of both giving and receiving feedback [8, 19, 39, 40]. Notably, both professions emphasised the significance of giving feedback to colleagues rather than receiving it themselves. They also found it valuable to receive aggregated data, such as unit- or hospital-wide compliance statistics and certificates from campaigns like "Aktion Saubere Hände". While previous studies have shown that hierarchical differences may discourage HCWs from speaking up when observing violations [41, 42], our interviewees indicated that hierarchical disparities or belonging to a different profession had no impact on their likelihood and willingness to provide feedback. This could be related to their confidence in their ability to perform correct hand hygiene and a culture of open communication and accountability in the workplace. However, it should be noted that many participants held leadership positions, which may have made it easier for them to provide feedback without facing negative consequences due to their status. Finally, we found that forming hand hygiene as a habit early on was suggested to be extremely beneficial for adherence. This has also been reported previously in other studies [43, 44], but we found that habit formation seemed to be especially relevant for physicians.

### Methodological, theoretical and practical implications

Conducting a fairly large amount of semi-structured interviews proved to be a good method for data collection as it gave participants the opportunity to elaborate on specific topics that they felt were particularly important. The TDF served as a solid foundation for this research, which was underlined by the fact that everything the participants mentioned was attributable to the TDF domains. However, the construct level of the revised version of the TDF [22] was not quite sufficient to code the content of the interviews. To ensure a complete analysis, some constructs from the original TDF [21] had to be reintroduced. For example, the constructs “habit” and "feedback", which were highly relevant to the content of the interviews, are not included in the revised TDF but were part of the original version. Furthermore, some constructs appear in more than one TDF domain and lack clear distinctions, which makes coding challenging. Consequently, it might be helpful for future research, if the TDF constructs were revised once again, possibly reintroducing some older constructs that were removed during the revision and defining clearer boundaries between domains.

Our findings highlight some important implications for the design of behaviour change interventions to improve hand hygiene compliance among HCWs and affirm existing approaches: First, good availability and proper placement of hand-rub dispensers make it easier for HCWs to comply with the hand hygiene guidelines [8, 15]. Moreover, placing signs might help to keep the behaviour salient and remind people of the importance of appropriate behaviour [8, 15, 38]. Second, it is essential to improve knowledge of the rationale for hand hygiene in healthcare settings and promote practical skills to follow the guidelines in daily practice through comprehensive on-the-job training [8, 15, 19]. The current training of medical students should be reconsidered, as the more theoretical teaching in university classes seems less effective than the practical instruction that nursing students receive during their vocational training. Additionally, it is essential to incorporate the “5 moments of hand hygiene” into the training of specific processes and procedures rather than teaching them outside of the contexts in which this information is relevant. This would ensure that HCWs learn the correct behaviour within their workflows, which should help to establish good habits early on, reducing both cognitive effort and the risk of error. Moreover, training must emphasise on the consequences of good and poor hand hygiene compliance for personal and patient safety, as these considerations can have a motivating effect [17]. Setting behavioural goals for oneself is also a common method in behaviour change interventions and can have a positive impact on the adherence of HCWs [9]. Finally, it is crucial to promote a culture of effective communication and positive error management. This might be achieved by establishing more regular compliance observations to provide immediate feedback on behaviours and guidance on how to optimise processes [8, 39, 40]. Moreover, HCWs should be encouraged to remind their colleagues about hand hygiene when they see omissions.

### Limitations

The current study also has several limitations. First, the close alignment of interview content with the TDF domains might be explained by the fact that the interview guide was based on the TDF. This naturally elicited responses relevant to the domains and might have hindered interviewees from elaborating more freely on facilitators for good hand hygiene compliance. Moreover, when asked about hygiene practices, interviewees may feel pressured to provide socially acceptable responses, especially if they feel evaluated. However, in this study, efforts were made to minimise this bias by using only open-ended questions and requesting concrete examples based on the interviewees’ real-life experiences. Additionally, the interviews were conducted by a neutral interviewer who was not part of the three hospitals’ infection prevention and control teams, helping to reduce perceived pressure. Another limitation is that the participant selection was based on their performance during compliance observation, which is only a brief behavioural example and could be biased by observational effects. Also, the selection of individuals for interviews did not take into account the quality of their hand hygiene. While most participants confirmed that they usually show high compliance, the statements of two interviewees suggested that their compliance record was rather mixed. Consequently, they were excluded from further data analysis. Another potential limitation of our study is that we did not aim to study the differences between types of wards and medical specialities despite some evidence that these exist (e.g., highest compliance usually in neonatal wards [45]). Consequently, we did not purposefully balance our sample for these aspects, resulting in a slightly skewed sample toward ICU staff (at least among nurses), which may have limited our ability to observe differences between ward types or speciality fields. Finally, while data was collected from HCWs working in three hospitals, all were located within the same region of Germany. Therefore, their experiences may not be applicable or generalisable to other regions, countries and healthcare settings.

## Conclusion

Overall, the findings of this study provide valuable insights into the factors helping to establish and sustain good hand hygiene compliance among physicians and nurses. The results highlight the importance of setting personal goals, habit development, comprehensive training, adequate environmental resources, and a positive communication culture in promoting adequate practices. The TDF was found to be a suitable model for identifying facilitators for hand hygiene behaviour among HCWs. By implementing new interventions or modifying already existing measures according to the results of the present study, we might be able to increase compliance with hand hygiene guidelines and thereby reduce rates of HAIs and improve patient safety.

## Supporting information

S1 File Codebook. (PDF)

S1 Table All codes with numbers. (XLSX)
