# Supplementary material for: Learning hand hygiene from the champions: Investigating key compliance facilitators among healthcare workers through interviews
Source: PLoS One. 2024 Dec 19;19(12):e0315456. doi: 10.1371/journal.pone.0315456 (PMC11658492; doi:10.1371/journal.pone.0315456)
Supplement: S1 File — (PDF) [file pone.0315456.s001.pdf]

TDF-Codebook

Color coding of the constructs / codes:

- **Codes / constructs from the TDF in the version of Cane et al. (2012)**
- **Codes / constructs inductively added or specifically created. Some of these are part of the TDF in the version of Michie et al. (2005)**

TDF Domain	Constructs and Codes	Definition	Guidance / Rules	Sample Quote
**Knowledge**<br><br>An awareness of the existence of something	**Knowledge**<br><br>• Knowledge	An awareness of the existence of something. Including knowledge of condition/scientific rationale	• Statements about the guidelines regarding proper hand hygiene in hospitals<br>• Knowledge of hospital acquired infections and relevant hospital germs<br>• Stating evidence-based reasons and consequences in favor of good hand hygiene / naming negative consequences as a result of poor hand hygiene (may overlap with the code "consequences, outcome expectancies, characteristics of outcome expectancies" from the domain *beliefs about consequences*)<br>• Knowledge of the importance of proper hand hygiene<br>• Statements, that knowledge of correct hand hygiene is important or that others should have the knowledge as well	*"With the background that nosocomial germs are of course a huge problem in hospitals and that the survival of patients is affected. In other words, I believe that a high standard of hygiene is an essential part of high-quality medicine."*
	**Procedural knowledge**<br><br>• Procedural knowledge	Knowing how to do something	• Descriptions of the process or procedure for proper hand disinfection / knowledge of how and when hand hygiene must take place	*"So essentially adhering to the five indications of hand hygiene: entering the working area or before contact with the patient, then after contact with the patient, when leaving the area, when preparing for or performing aseptic procedures, and after you've become potentially*

			• Naming one or more indications of the 5 moments of hand hygiene	*contaminated while handling secretions or materials of the patient."*
	**Knowledge of the task environment**<br><br>• Knowledge of the task environment<br>• During specific tasks<br>• Certain departments<br>• Specific type of patients	Knowledge of the social and material context in which a task is undertaken	• Statements on the relevance of good hand hygiene in a specific clinical setting, in dealing with specific patients or in executing specific clinical procedures	*"But every now and then you do eat food, but of course not in the controlled area. And it would be a good idea to disinfect your hands beforehand to prevent incorporation."*<br><br>*"When you take a dressing change or something else as an example: That's actually taking off the old dressing, then changing the gloves, disinfecting hands, putting on new gloves."*<br><br>*"But it really depends, so I would say that the understanding of hygiene is insanely high on the BMT ward. Because the knowledge of why you have to do it is simply there."*<br><br>*"What can also happen with our people is that if someone gets a multidrug-resistant germ and is severely immunosuppressed, then he already has a problem if he can no longer be treated, bone marrow transplanted or something like that."*
**Skills**<br><br>An ability or proficiency acquired through practice	**Skills assessment**<br><br>• Assessment of the skills of oneself and one's team<br>• Assessment of the skills of others	A judgment of the quality, worth, importance. Level or value of an ability or proficiency acquired through training and practice	• Statements in which the quality of one's own hand hygiene behavior or that of one's own team is evaluated negatively or positively	*"And I am sure the only reason they don't have that (a germ) is because we do it with care."*<br><br>*"There are physicians who work super hygienically just like nurses."*

	▪ Negative assessment<br>▪ Positive assessment		● Statements in which the quality of other people's hand hygiene behavior or their knowledge of it is evaluated negatively or positively	
	**Practice**<br><br>● Practice<br>● Lack of Practice	Repetition of an act, behaviour, or series of activities, often to improve performance or acquire a skill	● Statements on the influence of practice / training on hand hygiene behavior<br>● Statements about the relevance of practice / training in the correct performance of hand hygiene.	*"So it's actually trained behaviors."*<br><br>*"Yes, but as it is with content that you don't yet live so intensively in practice, it is perhaps difficult for students, because they don't have contact with the patients."*
	**Skills development**<br><br>● Skills development<br>● Different level of education<br>● Lack of offered training<br>● Differences between institutions<br>● Be taught / learned in everyday work or during induction<br>● Comparison of nurses and doctors regarding training in hand hygiene<br>● Further education (Link-Nurse, hygiene experts etc.)<br>● Training courses<br>● Study<br>▪ Insufficient<br>▪ Can not remember the study<br>▪ Not learned in the study<br>▪ Learned in the study<br>● Vocational training (nurses)<br>▪ Creating equal preconditions	The gradual acquisition or advancement through progressive stages of an ability or proficiency acquired through training and practice	● Setting in which the 5 moments of hand hygiene were learned / internalized<br>● Information on the development of the ability to perform hand hygiene correctly<br>● Statements about the quality of training regarding the correct hand hygiene	*"So it is, as I said, a constant learning process."*<br><br>*"I would say, there are primarily differences in education levels"*<br><br>*"Online training is good, but I think it would be better if this were somehow given a bit more prominence. That the topic would perhaps be promoted even more. I know that the online training is clicked through quickly, just so that one has it. Yes, [...] and then it's just difficult."*<br><br>*"It already appears that there are differences in the quality of the schools from which the nursing students come."*<br><br>*Where and how did you learn to perform and adhere to the five moments of hand hygiene at the hospital?*<br>*"[...]*And also through everyday work"

				“I believe that the nursing staff is much better at training than the medical staff. Because with the medical staff there is so much else that is also relevant, which I think is always neglected a bit in the routine and in the training. Or even with the physicians, there is always relatively little training per se. You read a lot about the main topics or what you think you have to learn. But with nursing, I think there are more learning modules in the basic education.”<br><br>*"In the course of it, it was actually that now I just recently became link-nurse, where I learned about the topic more in-depth. And I've been in the hospital for over 20 years now. I have always dealt with this topic because I was also a practice supervisor. So it was very, very important for me personally, because I also instructed the students and I had to know the basics. And that also lead to a in-depth understanding.“*<br><br>*"Yes, through further trainings, through specific hygiene trainings that take place on a regular basis. Once a year there is a hand disinfection training, also with black light, where you can see what you have reached and which areas you haven't."*<br><br>*"I'm not really sure anymore. I think it already started in the preclinic. So I think these five moments, that's something, that runs a bit through*

				the study, which you hear again and again. So I think in the infectiology lecture it was also a topic. Well, there are always lecturers who point it out to you during your studies, when you're with patients."<br><br>*"Yes, classically in the 3-year training. That's where you learned it in any case."*
	**Interpersonal skills**	An aptitude enabling a person to carry on effective relationships with others, such as an ability to cooperate, to assume appropriate social responsibilities or to exhibit adequate flexibility	Not coded	Not coded
	**Skills**	An ability or proficiency acquired through training and/or practice	Not coded	Not coded
	**Ability**	Competence or capacity to perform a physical or mental act. Ability may be either unlearned or acquired by education and practice	Not coded	Not coded
	**Competence**	One's repertoire of skills, and ability especially as it is applied to a task or set of tasks	Not coded	Not coded
**Social / professional role and identity**<br><br>A coherent set of behaviours and displayed personal qualities of an individual in a social or work setting	**Leadership**<br><br>• Leadership	The processes involved in leading others, including organising, directing, coordinating and motivating their efforts toward achievement of certain group or organization goals	• Influence of own leadership position / management function on own hand hygiene behavior or the behavior of others<br>• Information on actions to improve the hand hygiene behavior of others within the scope of one's own management position.<br>• Statements on the role model function with regard to hand	*"Maybe it has to do with being a practical instructor, that you are responsible for the practical training of the students. You then have a role model function and it's also more expected from you."*

			hygiene behavior based on the own leadership position (may overlap with the code "modeling" from the domain social influences)	
	**Social identity,** Identity, Group identity (Fusion)<br><br>● (Social / Group) Identity	The set of behavioural or personal characteristics by which an individual is recognizable [and portrays] as a member of a social group	● Statements of having responsibilities to others (outside of the hospital/profession).	*“Well, it's not just you, it's also your partner or your family at home. So that's another level that ultimately comes into play, because you're also responsible for them.”*
	**Identity**	An individual’s sense of self defined by<br>a) a set of physical and psychological characteristics that is not wholly shared with any other person and<br>b) a range of social and interpersonal affiliations (e.g., ethnicity) and social roles	Merged with the code „social identity” and coded there	Not coded here
	**Group identity**	The set of behavioural or personal characteristics by which an individual is recognizable [and portrays] as a member of a group	Merged with the code „social identity” and coded there	Not coded here
	**Professional role**<br><br>● Professional role<br>● Professional role of others<br>▪ Negative<br>▪ Positive	The behaviour considered appropriate for a particular kind of work or social position	● Good hand hygiene as an important, self-evident task of a physician / nurse (within the scope of the profession) (may overlap with the code "professional identity" from the same domain)<br>● Statements about correct hand hygiene behavior as part of professional responsibility<br>● Statements on the extent to which other employees see hand hygiene as part of their responsibility	*“We have a responsibility here [...] that we don't pass on pathogens.”*<br><br>*“I believe that all physicians and also all nurses are aware of the responsibility.”*

			(The statements must be related to hand hygiene behavior)	
	**Professional identity**<br><br>• Professional identity<br>• Problematic self-perception of others<br>• Ethics / moral	The characteristics by which an individual is recognized relating to, connected with or befitting a particular profession	• Good hand hygiene as an important, self-evident task of a physician / nurse (within the scope of the profession) (may overlap with the code "professional role" from the same domain)<br>• Good hand hygiene behaviour as part of one's own professional standards<br>• Lacking hand hygiene of others, due to problematic standards<br><br>(The statements that fit here must be related to the own person)	*“But [good hand hygiene] is actually my own ambition when it comes to patient care.”*<br><br>*“That they don't believe they were born sterile. Our surgeons in particular are always very, very keen to believe this.”*<br><br>*"[...] And I think you have to start the ethical discussions much earlier and say, how unethical is it actually to not disinfect your hands?"*
	**Professional confidence**	An individual’s belief in his or her repertoire of skills and ability especially as it is applied to a task or set of tasks	Solely coded in the domain *beliefs about capabilities*	Not coded here
	**Organizational commitment**	An employee’s dedication to an organization and wish to remain part of it	Not coded	Not coded
	**Professional boundaries**	The bounds or limits relating to, or connected with a particular profession or calling	Not coded	Not coded
**Beliefs about capabilities**<br><br>Acceptance of the truth, reality or validity about an ability, talent or facility that a person can put to	**Professional confidence,** Self-confidence (Fusion)<br><br>• (Professional) confidence	An individual’s beliefs in his or her repertoire of skills, and ability, especially as it is applied to a task or set of tasks.	• Statements about the quality, or value of one's own work force	*“Yes, so that others are doing better. If I'm not there, then someone else has to take over, who may not be quite as capable as I am. And from that point of view, I have a high priority to protect myself. Others may feel differently because they say, "Then I'll be sick." There exist completely different perceptions.“*

constructive use	**Self-confidence**	Self-assurance or trust in one's own abilities, capabilities and judgement	Merged with the code "professional confidence" and coded there	Not coded here
	**Self-esteem**	The degree to which the qualities and characteristics contained in one's self-concept are perceived to be positive	• Positive statements about one's own ability to comply with the 5 moments of hand hygiene	*"And I might have a little bit of a different standard there, but I would say my workforce is kind of important after all."*
	**Perceived behavioural control**<br><br>• Execution is difficult<br>▪ After touching a patient`s surroundings<br>▪ Before a procedure<br>▪ Before touching a patient<br><br>• Execution is easy<br>▪ After a procedure or body fluid exposure risk<br>▪ Before a procedure<br>▪ After touching a patient<br>▪ Before touching a patient	An individual's perception of the ease or difficulty of performing the behaviour of interest	• Indications of how difficult / easy it is for the interviewee to comply with the 5 moments of hand hygiene<br>• Indications for hand hygiene that are difficult or easy to comply with<br><br>(This code can overlap with the code "procedural knowledge" from the domain *knowledge*, if the indications are mentioned concretely)	*"Where I find it most difficult is before contact with patients. When you come out of the last room, when you walk past the dispenser, you disinfect your hands. That means I actually go into the next room with disinfected hands, and then when I re-disinfect my hands, I can't get into the glove. And that's always a big problem: freshly disinfected hands, they don't get in. So I think this point is actually the least one that gets realized."*<br><br>*"When it comes to activities on the patient, that is, before and after. In the care itself, in the basic body care I am also very certain."*
	**Self-efficacy,**<br>Perceived competence (Fusion)<br><br>• Self-efficacy / Perceived competence<br>• Lack of Self-efficacy / competence	An individual's capacity to act effectively to bring about desired results, as perceived by the individual	• Confidence in one's own ability to comply with the 5 moments / indications of hand hygiene<br><br>• Statements, that it is generally easy for the person to perform hand hygiene correctly<br><br>• Lack of confidence in one's own ability to comply with the 5 moments / indications of hand hygiene	*"And that's why there are no difficulties for me personally."*<br><br>*"But we only hinder ourselves, circumstances around not really."*
	**Perceived competence**	An individual's belief in their ability to learn and execute skills	Merged with the code "self-efficacy" and coded there	Not coded here

	**Beliefs**	The thing believed; the proposition / set of propositions held true	Not coded	Not coded
	**Empowerment**	The promotion of the skills, knowledge and confidence necessary to take great control of one's life as in certain educational or social schemes; the delegation of increase decision-making powers to individuals or groups in a society or organization	Not coded	Not coded
**Optimismus**<br><br>The confidence that things will happen for the best or that desired goals will be attained	**Pessimism**<br><br>• Pessimism	The attitude that things will go wrong and that people's wishes or aims are unlikely to be fulfilled	• Pessimistic views, regarding long-term improvement of hand hygiene compliance in hospitals and reasons given for own pessimism	*"Well, I'm not optimistic. And I would be happy if we could keep it at the level it is now. There's certainly always room for improvement, but everyday work is becoming increasingly stressful and staffing numbers aren't getting much bigger. And that's why I think things like hand hygiene may be somewhat neglected. That much about pessimism."*
	**Optimism**<br><br>• Optimism	The attitude that outcomes will be positive and that people's wishes or aims will be ultimately fulfilled	• Optimistic views, regarding long-term improvement of hand hygiene compliance in hospitals and reasons given for own optimism	*„Generally optimistic, in fact. I think this new generation in particular is paying more attention to this, especially in terms of the last Corona years. I think there's been a bit of a change. Yeah, so i am optimistic."*
	**Unrealistic Optimism**	The inert tendency for humans to over-rate their own abilities and chances of positive outcomes compared to those of other people	Not coded	Not coded
**Beliefs about consequences**<br><br>Acceptance of the	**Consequences,** Outcome expectancies, Characteristics of outcome expectancies (Fusion)	An outcome of behaviour in a given situation	• Assessments of the expected consequences of good or poor hand hygiene. The consequences must be related	*"If I don't behave correctly in that regard, then yes, I may have done something great with a great surgery or a great procedure or aiding the patient, but I may have*

truth, reality or validity about outcomes of a behaviour in a given situation	• Negative consequences<br>• Positive consequences		to hand hygiene behavior (May overlap with the code "knowledge" from the domain *knowledge*)	*created a huge amount of damage if I didn't follow hand hygiene."*<br><br>*"[...]Of course you are protected when you disinfect your hands."*
	**Outcome expectancies**	Cognitive, emotional, behavioural, and affective outcomes that are assumed to be associated with future or intended behaviour. These assumed outcomes can either promote or inhibit future behaviours	Merged with the code „consequences" and coded there	Not coded here
	**Characteristics of outcome expectancies**	Characteristics of the cognitive, emotional and behavioural outcomes that individuals believe are associated with future or intended behaviours and that are believed to either promote or inhibit these behaviours. These include whether they are sanctions/rewards, proximal/distal, valued/not valued, probable/improbable, salient/not salient, perceived risks or threats	Merged with the code „consequences" and coded there	Not coded here
	**Anticipated regret**<br><br>• Anticipated regret	A sense of the potential negative consequences of a decision that influences the choice made: for example an individual may decide not to make an investment because of the feelings associated with an imagined loss	• In order to avoid the possible feeling of remorse, which could occur in case of non-compliance with hand hygiene, hand hygiene is performed as correctly as possible beforehand	*"And of course the protection for the next patient, that I am not the reason for carrying on any germ or any bacteria."*
	**Beliefs**<br><br>• Beliefs<br>• Hardly any risk to self	The thing believed; the proposition or set of propositions held true	• Beliefs about the consequences of poor/good hand hygiene (especially strong opinions about the possible consequences) (May overlap with the code "consequences, outcome expectancies, characteristics of	*"Yes, hand hygiene saves lives."*<br><br>*"I mean, if there's a germ on my skin now, it's certainly not going to kill me."*

			outcome expectancies" from the same domain<br>• Belief that lack of hand hygiene does not put one at great risk / oneself at lower risk of negative consequences	
**Reinforcement**<br><br>Increasing the probability of a response by arranging a dependent relationship, or contingency, between the response and a given stimulus	**Rewards**<br><br>• No rewards	Return or recompense made to, or received by a person contingent on some performance	• Statements about receiving or not receiving a reward in the sense of a quid pro quo for the correct performance of hand hygiene.<br><br>(If the reward is praise, the statement is to be coded within the same domain with the code "positive consequences")	*"So actually I wouldn't have heard anything that it would be rewarded in any way."*
	**Punishment,**<br>Sanctions (Fusion)<br><br>• Punishment / Sanctions<br>• No punishment / Sanctions<br>• Punishment / Sanctions do not help	The process in which the relationship between as response and some stimulus or circumstance results in the response becoming less probable; a painful, unwanted or undesired event or circumstance imposed as a penalty on a wrongdoer	• Statements on receiving or not receiving punishment for not performing hand hygiene correctly<br><br>(If the punishment is reprimand, the statement is to be coded within the same domain with the code "negative consequences")	*"We actually have had the case in my old hospital, if people did not adhere to it and we pointed it out several times, we send the hygiene experts over. They were then given special training, and if that didn't work either, they were sometimes threatened with a warning. So that also occurs."*<br><br>*"So to my knowledge it is not currently punished at all."*<br><br>*"Yes, I believe that bans or sanctions usually don't get you anywhere."*
	**Sanctions**	A punishment or other coercive measure, usually administered by a recognized authority, that is used to penalise and deter inappropriate or unauthorized actions	Merged with the code "punishment" and coded there	Not coded here

	**Consequences**<br><br>• Negative consequences<br>• Positive consequences	An outcome of behaviour in a given situation	• Consequences for the person themself and / or their colleagues. Furthermore the stated consequences must be imposed on one from the outside / by someone else (e.g. in the sense of a praise / reprimand)<br><br>(Not the same as the codes "reward" and "punishment" from the same domain).<br><br>(May overlap with codes "feedback" from the domains *social influences* and *behavioral regulation*)<br><br>(This code is to be clearly distinguished from the code "consequences " from the domain *beliefs about consequences*)	*"Incorrect execution punished in the sense that if there's a inspection that you get a slap on the wrist."*<br><br>*"Rewarded? Praise for doing your job adequately or for your hand hygiene."*
	**Incentives**<br><br>• Incentives	An external stimulus, such as condition or object, that enhances or serves as a motive for behaviour	• Statements on the relevance of incentives in hand hygiene compliance<br>• Existing / possible incentives that promote hand hygiene behavior	*"You could have a little competition like that, for example: who uses the most disinfectant? [...]."*
	**Reinforcement**<br><br>• Reinforcement	A process in which the frequency of a response is increased by a dependent relationship or contingency with a stimulus		*"Yes, you always have to consider how can it ultimately be positively reinforced."*
	**Contingencies**	A conditional probabilistic relation between two events. Contingencies may be arranged via dependencies or they may emerge by accident	Not coded	Not coded

**Intentions**<br><br>A conscious decision to perform a behaviour or a resolve to act in a certain way	**Intentions** (Michie et al. 2005)<br><br>● Intentions	A conscious decision to perform a behaviour or a resolve to act in a certain way	● Statements on the conscious decision / the determination to carry out hand hygiene in the daily work routine	*“So it's a conscious decision. On an abstracted level, to apply these five moments of hand hygiene. That is a conscious decision..“*
	**Stability of intentions**<br><br>● Stability of intentions	The ability of one’s resolve to remain in spite of disturbing influences	● The ability / willingness to comply with the 5 moments of hand hygiene despite disruptive influences	*“So even if I'm having trouble with skin tolerance, I still continue to disinfect my hands.”*
	**Transtheoretical model and stages of change**	A five-stage theory to explain changes in people’s health behaviour. It suggests that change takes time, that different interventions are effective at different stages, and that there are multiple outcomes occurring across the stages	Not coded	Not coded
	**Stages of Change model**	A model that proposes that behaviour change is accomplished through five specific stages	Not coded	Not coded
**Goals**<br><br>Mental representations of outcomes or end states that an individual wants to achieve	**Goal priority**<br><br>● Goal priority	Order of importance or urgency of end state toward which one is striving	● Statements regarding (the order of) the importance of the intended goals, which are to be achieved with correct hand hygiene<br><br>● Statements about proper hand hygiene as a priority	*"I don't want to kill my patients. That's just the ultimate reason.”*
	**Goals (proximal/distal),** Goals (autonomous/controlled) (Fusion)<br><br>● Hand hygiene becomes a habit<br>● Protection of others<br>▪ Protection of relatives	Desired state of affairs of a person or system, these may be closer (proximal) or further away (distal)	● Statements about what the interviewees want to achieve with / in adhering to the 5 moments of hand hygiene / what motivates them to adhere to them	*“And that at some point, it turns into an almost automated and unconscious process. That would actually be something that might be desirable.”*<br><br>*“And in this sense, it is of course very, very important that we do not*

	▪ Protection / well-being of patients<br>▪ Protection of colleagues<br>● Correct performance of hand hygiene as goal<br>● Self-protection / own well-being<br>● Avoid transmission of germs		(The stated goals must relate to hand hygiene behavior. Other goals of the respondent are not to be coded)	*endanger our patients with unhygienic practices."*<br><br>*"And that the goal then for execution is to adhere to it in as many moments as possible."*<br><br>*"And therefore my aspiration to protect myself is strong."*<br><br>*"Because, after all, I don't want to contribute to anything being passed on in terms of infections or that I'm harming the patient."*
	**Goals (autonomous/controlled)**	The end state toward which one is striving: the purpose of an activity or endeavour. It can be identified by observing that a person ceases or changes their behaviour upon attaining this state; proficiency in a task to be achieved within a set period of time	Merged with the code "goals (distal/proximal)" and coded there	Not coded here
	**Action planning**	The action or process of forming a plan regarding a thing to be done or a deed	Solely coded in the domain *behavioural regulation*	Not coded here
	**Implementation intention**	The plan that one creates in advance of when, where and how one will enact a behaviour	Solely coded in the domain *behavioural regulation*	Not coded here
	**Goal / target setting**	A process that establishes specific time based behavioural targets that are measureable, achievable and realistic	Not coded	Not coded
**Memory, attention and decision processes**<br><br>The ability to retain information, focus	**Cognitive overload / tiredness**<br><br>● Cognitive overload / tiredness	The situation in which the demands placed on a person by mental work are greater than a person's mental abilities	● Situations in which the person's own abilities are not sufficient to perform hand hygiene correctly because the person is	*"Yes, sometimes it's not that easy, I think. When there are stressful situations or when there's just a lot going on and your mind is already ten steps ahead."*

selectively on aspects of the environment and choose between two or more alternatives			cognitively burdened / overloaded with other things	
	**Decision making**<br><br>• Decision making	The cognitive process of choosing between two or more alternatives, ranging from the relatively clear-cut to the complex	• Details on situations where the person must choose between proper hand hygiene and something else and actively chooses one over the other	*"And I can well imagine that first and foremost the vital threat is mentally in the foreground and hand disinfection is in the background. And I think that's a evaluation of risks in this case."*
	**Attention,**<br>Attention control (Fusion)<br><br>• Be attentive to it yourself<br>• Draw attention to it<br>• Distracting factors	A state of awareness in which the senses are focused selectively on aspects of the environment and the central nervous system is in a state of readiness to respond to stimuli	• Statements to focus oneself on proper hand hygiene and to perform it attentively / consciously<br>• External factors that draw one's attention to hand hygiene / make it present<br>• Factors that divert attention away from proper hand hygiene	*"Yes, for example you do it maybe more consciously before going on your break and you go to lunch. I do it even more consciously, when I'm changing bandages."*<br><br>*"We did the compliance observations. We put up posters on how hand disinfection is done and why it is important. This is already a sustained topic and also very present in our facility. [...]"*<br><br>*"Now, if there are extremely high volumes and alarms everywhere, it's rather difficult."*
	**Attention control**	The extent to which a person can concentrate on relevant cues and ignore all irrelevant cues in a given situation	Merged with code „attention" and coded there	Not coded here
	**Memory**<br><br>• Memory<br>• Forgotten theory about hand hygiene<br>• Execution forgotten<br>▪ After touching a patient´s surroundings<br>▪ Before a procedure<br>▪ After touching a patient<br>▪ Before touching a patient<br>• Remember the execution	The ability to retain information or a representation of a past experience, based on the mental processes of learning or encoding retention across some interval of time, and retrieval or reactivation of the memory; specific information of a specific task	• The person is not able to remember and enumerate all 5 moments of hand hygiene in wording<br>• Statements, whether the 5 moments (or some indications of it) are sometimes forgotten to comply with<br>• Statements whether it is easy to remember to perform the 5 moments of hand hygiene (some indications of it)	*"It's not as if the employees don't have any theoretical knowledge. It just means it has to be sustained. It has to be like hypnosis, becoming a recurring subject over and over again."*<br><br>*"If it were a matter of listing all five moments in detail, I would possibly have difficulties."*<br><br>*"That's when it can go under sometimes, when you're somehow*

	▪ After a procedure or body fluid exposure risk<br>▪ Before a procedure<br>▪ Before touching a patient<br>▪ After touching a patient<br>▪ After leaving the patient´s room		(For named indications of hand hygiene, there may be overlaps with the code "procedural knowledge" from the domain *knowledge*)<br><br>(Can sometimes not be clearly separated from the codes "Execution is easy / difficult" from the domain *beliefs about capabilities*)	*already called that you have to go to the OR, but you are still in the last patient room. Then it's not so easy to remember, sometimes."*<br><br>*"But I think you can definitely implement it well and then it's easy to remember."*
**Environmental context and resources**<br><br>Any circumstance of a person's situation or environment that discourages or encourages the development of skills and abilities, independence, social competence and adaptive behaviour	**Barriers and facilitators**<br><br>● Facilitators<br>▪ Special pathogen<br>▪ Skin protection<br>▪ Easy / specific patients<br>▪ Education, continuing education, training etc.<br>▪ Resources / material resources<br>▪ Good tolerance of disinfectants<br>▪ Sufficient personnel<br>▪ Availability of time<br>▪ Availability of desinfectant<br>▪ Salient events / critical incidents<br>▪ Corona-virus pandemic<br>● Barriers<br>▪ Pathogen not visible<br>▪ Much personnel turnover<br>▪ No skin protection<br>▪ Specific pathogens<br>▪ Corona-virus pandemic	In psychological contexts, barriers/ facilitators are mental, emotional or behavioural limitations/ strengths in individuals or groups	● Environmental factors / external or structural influences that promote or hinder compliance with the 5 moments of hand hygiene<br>● Resources (material or human) which are necessary for good hand hygiene behavior / which, if lacking negatively influence behavior.<br>● Indications that certain environmental factors are not a barrier to hand hygiene behavior or are not a problem<br>● Prominent and concretely described events in connection with the 5 moments of hand hygiene<br>● External factors that cause stress and thus influence hand hygiene behavior	*So certainly conducive is the good availability. As I said, we actually have the dispensers mounted everywhere.*<br><br>*"And the hindrance [...] is primarily the work density and the time pressure [...]."*<br><br>*"We don't have any difficulties with deliverability [of disinfectant] now and with availability as well."*

	▪ Gloves<br>▪ Resources / material Resources<br>▪ Bad tolerance of desinfectants<br>▪ Poor availybility of desinfectants<br>▪ Lack of personnel<br>▪ Lack of time<br>▪ Environmental stressors<br>▪ Emergencies<br>▪ Rounds<br>● No barriers<br>▪ Tolerance is no problem<br>▪ Lack of time is no barrier<br>▪ Availability is no problem			
	**Resources / material resources**	Commodities and human resources used in enacting a behaviour	Integrated into the codes “barriers” and “facilitators”	Not coded here
	**Salient events / critical incidents**	Occurrences that one judges to be distinctive, prominent or otherwise significant	Integrated into the codes “barriers” and “facilitators”	Not coded here
	**Environmental stressors**	External factors in the environment that cause stress	Integrated into the codes “barriers” and “facilitators”	Not coded here
	**Person x environment interaction**<br>● Person x environment interaction	Interplay between the individual and their surroundings	● Interaction between the person and their environment, which impacts compliance with the 5 Moments of Hand Hygiene (e.g. disinfectant dispensers, signs, etc.)	*"And what I also liked are these pictures from the Campaign “Aktion Saubere Hände", where you can also see these graphics [...]."*
	**Organizational culture / climate**	A distinctive pattern of thought and behaviour shared by members of the same organization and reflected in their language, values, attitudes, beliefs and customs	Not coded	Not coded

**Social influences**<br><br>Those interpersonal processes that can cause individuals to change their thoughts, feelings or behaviours	**Reciprocity**<br><br>● Reciprocity	The quality of an act, process, or relationship in which one person receives benefits from another and, in return, provides an equivalent benefit	● Statements to maintain proper hand hygiene and expect others to do the same in return	*"They of course expect that we maintain absolutely proper hygiene, and that's beneficial, of course. On the other hand, we expect the same from our patients."*
	**Empathy**<br><br>● Empathy	Understanding a person from his or her frame of reference rather than one's own, or vicariously experiencing that person's feelings, perceptions, and thoughts. Empathy does not, of itself, entail motivation to be of assistance, although it may turn into sympathy or personal distress, which may result in action. […]	● Putting oneself in the position of the patient, which results into the willingness to perform hand hygiene correctly	*"I would also like to see absolute cleanliness maintained if I were a patient myself."*
	**Supervision** (Michie et al. 2005)<br><br>● Supervision<br>● No observation	Oversight: critical evaluation and guidance provided by a qualified and experienced person (the supervisor) to another individual (the trainee) during the learning of a task or process. […]	● Statements on compliance observations<br>● Statements about the (lack of) observation during hand disinfection (also by colleagues or patients)<br>● Graded examinations as part of the training (in the sense of monitoring proper hand hygiene)	*"And these observations are very important. When there are hygiene experts on the ward, then this helps you. [...]"*<br><br>*"So honestly, I don't think anyone is watching me and my hands."*
	**Team Work / Collaboration** (Michie et al. 2005)<br><br>● Team Work / Collaboration	The act or process of two or more people working together to obtain an outcome desired by all […]	● Statements about the collaboration of colleagues with the aim of complying with hand hygiene	*"We try to have a positive culture in the team and point out hygiene mistakes to each other or point out good hygiene behaviour as well"*
	**Hierarchy** (Michie et al. 2005) **/ Different Profession**<br>● Not relevant with feedback<br>● Relevant with feedback	A clear ordering of individuals on some behavioral dimension, such as dominance–submission. A linear hierarchy occurs when all individuals can be arrayed in a strict transitive order along a continuum. Often, however, a hierarchy is more complex, with some individuals having equal status or acting in coalitions, or	● The influence of hierarchical structures on hand hygiene behavior<br>● The relevance of the professional position / status in giving feedback on hand hygiene behavior	*"It doesn't make any difference in the context of their profession, because I would tell everyone, so from the head physician to the cleaning staff. If it's just not correct or you can improve it, then I think it's fine to address that."*<br><br>*"Yes, but the physicians don't approach their attending at the door*

		with ordering based on different factors		*and tell her, that she didn't disinfect her hands when she left the room."*
	**Feedback** (Michie et al. 2005)<br><br>● Feedback<br>● Getting feedback<br>● Getting no feedback<br>● Giving feedback<br>● Giving no feedback	Information about a process or interaction provided to the governing system or agent and used to make adjustments that eliminate problems or otherwise optimize functioning. It may be stabilizing negative feedback or amplifying positive feedback [...]	● Statements to give feedback / communicate to each other regarding hand hygiene behavior.<br>● Statements about giving feedback to other employees about their hand hygiene behavior<br>● Statements about receiving feedback regarding one's own hand hygiene behavior from other people (must have been given directly by a person)<br><br>(Always to be double coded with the code "feedback" from the domain behavior regulation)<br><br>(May overlap with the code "positive / negative consequences" from the domain reinforcement)	*"Yes, that you point this out to each other. Now, for example, just when you've touched an isolated patient or also approach the general care trolley with hands that haven't been disinfected, that you point it out to each other."*<br><br>*"Well, there are always lecturers who point it out to you during your studies when you're with patients."*<br><br>*"But in everyday work, no one is going to say, 'You did that particularly well now.'"*<br><br>*"If I notice something, I address it. If it's a more serious hygienic mistake, then I address that [...]."*<br><br>*"But I'm honest, if the patient doesn't have a germ, then of course you have to do hand disinfection in the same way. You don't remind anyone of this, no matter which professional group. I rarely say to my own nursing colleagues:´you know, you have to disinfect your hands again.´ And I won't say it to my medical colleagues either. Yes, I'm honest about that."*
	**Modeling**<br><br>● Modeling	The process in which one or more individuals or other entities serve as examples (models), that [a person] will emulate […]	● Relevance of observation and imitation in hand hygiene behavior<br>● Statements on own function as a role model with regard to correct hand hygiene	*"And I think roll modeling is a key aspect that supports it. Just demonstrating a conscious hygienic culture."*

			• The relevance of role models in adherence to the 5 moments of hand hygiene	
	**Intergroup conflict**<br><br>• Conflict	Disagreement or confrontation between two or more groups and their members. This may involve physical violence, interpersonal discord, or psychological tension	• Emergence of conflicts, disagreements between people concerning hand hygiene behavior	*"Yes, in principle I would say that the tone makes the music. That's very important, because if you point out to someone who has been working on the ward for an extremely long time that he's been performing hand disinfection incorrectly, then that's actually guaranteed not to go down well and is more likely to cause a conflict. And yes, they would find it disrespectful if a beginner were to accuse them of something."*
	**Power**<br><br>• Power<br>• Lack of power	The capacity to influence others, even when they try to resist this influence	• Statements that one is (not) able to influence other persons regarding their hand hygiene behavior	*"That is, if I care for a patient with a colleague [...] that will also influence [behavior] positively."*<br><br>*"I'm honest now, I don't think [I have] that much of an impact. I don't think so, actually."*
	**Social support**<br><br>• Social support	The apperception or provision of assistance or comfort to others, typically in order to help them cope with a variety of biological, psychological and social stressors. Support may arise from any interpersonal relationship in an individual's social network, involving friends, neighbours, religious institutions, colleagues, caregivers of support groups	• The provision of assistance / support by others, which is conducive to compliance with the 5 moments of hand hygiene	*"Yes, so the positive reinforcement among each other in the team is certainly beneficial. Where everybody knows that it is important. So that's mutual positive reinforcement."*
	**Group norms,**<br>Social norms (Fusion)<br><br>• Group / social norms	Any behaviour, belief, attitude or emotional reaction held to be correct or acceptable by a given group in society	• Proper hand hygiene as a behavior that is considered correct and important among the group of hospital employees	*"I think it's quite important because it just makes a very different appearance to the outside world."*
	**Social norms**	Socially determined consensual standards that indicate a) what behaviours are considered typical	Merged with the code "group norms" and coded there	Not coded here

		in a given context and b) what behaviours are considered proper in the context		
	**Social comparisons**	The process by which people evaluate their attitudes, abilities or performance relative to others	• Observing other employees and comparing their hand hygiene behaviors with yours	*"[...] I sometimes watch my colleagues. To see how they do it, if maybe I can do it too, to do be better at it. [...]"*
	**Group conformity**	The act of consciously maintaining a certain degree of similarity to those in your general social circles	• Adherence to the 5 moments of hand hygiene in order to resemble the other employees / colleagues or not to be noticed in a negative way.	*"[...] you actually want to fit into your environment, so to speak [...]."*
	**Social pressure**<br><br>• Social pressure<br>• No social pressure / no influence by others	The exertion of influence on a person or group by another person or group	• Being influenced regarding hand hygiene behavior by other people (positively or negatively)<br>• Not being influenced regarding hand hygiene behavior by other people (positively or negatively	*"I think it's important that patients have expectations. And it would be embarrassing for me personally if I were asked by patients that I should disinfect my hands. But that is good and that is certainly something that is also important for younger colleagues, an important incentive. The other way around? So if a colleague were to approach me now: ´Why didn't you disinfect your hands?´ Of course, that would also be a point for me where I would say: ´I have to work on myself.´"*<br><br>*"So the expectation from the patients deliberately has no direct influence."*
	**Group identity**	The set of behavioural or personal characteristics by which an individual is recognizable [and portrays] as a member of a group	Solely coded in the domain *social influences*	Not coded here
	**Alienation**	Estrangement from one's social group; a deep seated sense of dissatisfaction with one's personal experiences that can be a source of lack of trust in one's social or physical environment or in oneself;	Not coded	Not coded

		the experience of separation between thoughts and		
**Emotion**<br><br>A complex reaction pattern, involving experiential, behavioural and physiological elements, by which the individual attempts to deal with a personally significant matter or event	**Emotion**<br><br>• Emotions are relevant<br>• Emotions are not relevant	A complex reaction pattern, involving experiential, behavioural and physiological elements, by which the individual attempts to deal with a personally significant matter or event	• Statements that emotions play a role or no role in adherence to the 5 moments of hand hygiene	*"Overall, of course, it can certainly play a role in other situations, for example, in the emergency service. Yesterday night, I was in the middle of a very severe hemorrhage. Then, of course, the emotions play a greater role, so that you pay even closer attention to hygiene. If you don't know what the patient has and then a large amount of blood is involved. So it certainly plays a role there."*<br><br>*"But other than that, I don't think it's a very emotional topic."*
	**Inner need**<br><br>• Inner need	A condition of tension in an organism resulting from deprivation of something required for survival, well-being, or personal fulfillment	• Stating the inner need to disinfect the hands. Or to have the feeling that something is missing if you do not do it	*"I think then it's always harder to implement or think of than after contact with a patient. That's when you tend to feel the need to disinfect your hands anyway."*
	**Positive / negative affect**<br><br>• Positive / negative affect	The internal feeling/state that occurs when a goal has/has not been attained. A source of threat has/has not been avoided, or the individual is/is not satisfied with the present state of affairs	• Description of the feeling that occurs with successful compliance or non-compliance with hand hygiene.	*"And I'd say for my own comfort it also plays a big role, because with disinfected hands you feel much more comfortable on the ward."*
	**Stress**<br><br>• Stress	A state of physiological or psychological response to internal or external stressors	• Statements that feeling stressed has an influence on hand hygiene behavior<br><br>(Not to be coded for descriptions of stressful situations if the feeling of stress is not explicitly mentioned)	*"When I look at the basic general attitude of nursing staff, how they sometimes don't care about anything because they're just overworked, stressed, don't have time. That's why I see it quite pessimistically, that things can be forgotten or that you just don't feel like doing it."*

	**Affect**<br><br>• Affect	An experience or feeling of emotion, ranging from suffering to elation, from the simplest to the most complex sensations of feelings, and from the most normal to the most pathological emotional reactions	• The influence of one's own mood or emotional state on the hand hygiene behavior	*"So I think maybe personal mood plays into it as well. That can influence it quite a bit, yes."*
	**Guilt**<br><br>• Guilt is not relevant<br>• Guilt is relevant	A self-conscious emotion characterized by a painful appraisal of having done (or thought) something that is wrong and often by a readiness to take action designed to undo or mitigate this wrong […]	• Statements on the relevance of guilt / conscience in adherence to the 5 moments of hand hygiene	*"Emotions like guilt don't play a role now either. So then I would have to have done something wrong before to feel guilt now. No."*<br><br>*"I would understand guilt maybe even more. Because I might think to myself, if I don't do it right, I might endanger someone."*
	**Disgust**<br><br>• Disgust is relevant	A strong aversion, for example, to the taste, smell, or touch of something deemed revolting, or toward a person or behavior deemed morally repugnant	• Statements on the relevance of disgust in adherence to the 5 moments of hand hygiene	*"Yeah, I would put disgust at the top of the list. So that's, I think, the biggest impetus for someone to immediately do massive cleaning and disinfection. [...]"*
	**Fear,**<br>Anxiety (Fusion)<br><br>• Fear is relevant<br>• Fear is not relevant	An intense emotion aroused by the detection of imminent threat, involving an immediate alarm reaction that mobilizes the organism by triggering a set of physiological changes	• Statements on the relevance of fear in adherence to the 5 moments of hand hygiene	*"But that may be a slight fear of contagion as a motivator."*<br><br>*"Well yes, but I'm not afraid, it's just part of the routine".*
	**Anxiety**	A mood state characterized by apprehension and somatic symptoms of tension in which an individual anticipates impending danger, catastrophe or misfortune	Merged with the code "fear" and coded there	Not coded here
	**Burnout**	Physical, emotional or mental exhaustion, especially in one's job or career, accompanied by decreased motivation, lowered performance and negative attitudes towards oneself and others	Not coded	Not coded

	**Depression**	A mental state that presents with depressed mood, loss of interest or pleasure, feelings of guilt or low self-worth, disturbed sleep or appetite, low energy, and poor concentration	Not coded	Not coded
**Behavioural regulation**<br><br>Anything aimed at managing or changing objectively observed or measured actions	**Action planning,** Implementation intention (Fusion)<br><br>• Action planning / Implementation intention<br>• Lack of action planning	The action or process of forming a plan regarding a thing to be done or a deed	• Descriptions or the lack of strategies, plans, ways to maintain proper hand hygiene	*"So concrete strategies help for sure. And that you simply make a resolution to disinfect your hands every time you go into the patient's room, regardless of whether you are doing something or not. And I think something like that actually helps."*<br><br>*"And especially it's sometimes during rounds where you don't necessarily plan to touch the patient and then you're just standing by the bed and in the end you do lift the covers after all."*
	**Implementing interventions / Change-Management** (Michie et al. 2005)<br><br>• Implementing interventions / Change-Management	The process of planning, implementing, and evaluating change within organizations or communities, with the goal of realizing the benefits of change while implementing it as efficiently, smoothly, and cost-effectively as possible. Change management practices include effective communication regarding the change, such as the identification of intended consequences, and the prediction of and preparation for unintended consequences	• Information on interventions or events that focus on correct hand hygiene and change behavior for the better	*"We did the compliance observations. We put up posters on how hand disinfection is done and why it is important. This is already a sustained topic and also very present in our facility. And therefore I could deepen that quite well."*
	**Workflow**<br><br>• Workflow	The way that a particular type of work is organized, or the order of the stages in a particular work process	• The influence of one's workflow / process in adhering to the 5 moments of hand hygiene or when it is disrupted	*"So I think it's totally important to have set workflows and also have set ways of doing things."*

	● Workflow disrupted / unfamiliar work process	(This definition is taken from the Cambridge Dictionary, as there is no definition for the term "workflow" in the APA Dictionary of Psychology)	● The influence of standardized procedures in adherence to the 5 moments of hand hygiene<br>● Explanation of one's own workflow with regard to proper hand hygiene	*"But it's just these interruptions. When a patient has to go to an examination, it has to be done quickly, quickly, quickly. And then you forget to take three plugs with you or you have to do something on the central venous line. And those are the moments where you probably get a little sloppy."*
	**Feedback** (Michie et al. 2005)<br><br>● Feedback<br>● Getting feedback<br>● Getting no feedback<br>● Giving feedback<br>● Giving no feedback	Information about a process or interaction provided to the governing system or agent and used to make adjustments that eliminate problems or otherwise optimize functioning. It may be stabilizing negative feedback or amplifying positive feedback [...]	● Statements to give feedback / communicate about hand hygiene behavior to each other<br>● Statements about giving feedback to other employees about their hand hygiene behavior<br>● Statements about receiving feedback regarding one's own hand hygiene behavior<br><br>(Does not have to be given directly by a person, e.g. in the form of certificates, statistics)<br><br>(May overlap with the codes "negative / positive consequences" from the domain *reinforcement*)<br><br>(Only code simultaneously with the code "feedback" from the domain *social influences*, if the feedback comes directly from a person)	*"Yeah, so I think it's extremely important. [...] I would call someone out on it if they don't do it, but at the same time I expect them to call me out if I don't do it."*<br><br>*"And [patients] do pay attention to whether you disinfect your hands or in very rare cases [...] also approach the staff and say: ‘you didn't disinfect your hands before you examined me or did anything on me.´"*<br><br>*"You actually don't get a lot of praise. I don't know if many people pay attention to it [...]. In any case, at least in the time I've been there, I've never really received positive feedback that was so explicitly expressed [...]."*<br><br>*"Because every hospital and clinic has its own hygiene supervisor, and there are things we see and criticize on a regular basis."*<br><br>*"But I'm honest, if the patient doesn't have a germ, then of course you have to do hand disinfection in the same way. You don't remind anyone of this, no matter which professional group. I rarely say to*

				my own nursing colleagues:´you know, you have to disinfect your hands again.´ And I won't say it to my medical colleagues either. Yes, I'm honest about that."
	**Habit** (aus Michie et al. 2005)<br><br>● Habit<br>▪ Negative influence<br>▪ Positive influence<br>● Lack of routine / no habit	A well-learned behavior or automatic sequence of behaviors that is relatively situation specific and over time has become motorically reflexive and independent of motivational or cognitive influence—that is, it is performed with little or no conscious intent. For example, the act of hair twirling may eventually occur without the individual's conscious awareness	● Influence of routine / habit on adherence to the 5 moments of hand hygiene<br>● Statements about how much the 5 moments of hand hygiene are internalized / routine	*"[...] So since it's second nature to me, it's completely normal for me."*<br><br>*"But those are the kinds of things that, if you don't do it as much anymore, or even if you don't think about it at all, then sometimes you're already insecure about it."*
	**Breaking habit**<br><br>● Breaking habit	To discontinue a behaviour or sequence of behaviours that is automatically activated by relevant situational cues.	● Statements on wanting to break / having broken certain habits to change hand hygiene behavior	*"[...] and the negative habits must eventually be corrected over and over again."*
	**Self-monitoring**<br><br>● Self-monitoring	A method used in behavioural management in which individuals keep a record of their behaviour, especially in connection with efforts to changes or regulate the self; a personality trait reflecting an ability to modify one's behaviour in response to a situation	● Self-reflection of one's own hand hygiene behavior in order to improve it<br>● Self-monitoring of hand hygiene behavior	*"But as I said, I think you still have to keep in mind: What am I doing exactly? Am I still doing this properly or is this hand sanitizing just pro forma? Or is it right, what I should be doing."*
	**Incomprehensible / unassignable**			
